# Active surveillance cultures and cohorting for carbapenem-resistant *Acinetobacter baumannii* in an endemic setting: an interrupted time-series analysis

**DOI:** 10.1017/ice.2026.10427

**Published:** 2026-06

**Authors:** Regev Cohen, Shelly Lipman-Arens, Orna Ben-Natan, Aliza Vaknin, Mohammed Ganayem, Yael Galnoor Tene, Linor Ishay, Olga Simon Feld, Milena Pitashny, Alvira Zbiger, Rena Abilevitch, Said Younis, Elias Tannous

**Affiliations:** 1 Faculty of Medicine, Technion-Israel Institute of Technology, Haifa, Israel; 2 Infection Control Unit, Hillel Yaffe Medical Center, Hadera, Israel; 3 Infectious Diseases Unit, Hillel Yaffe Medical Center, Israel; 4 Microbiology Laboratory, Hillel Yaffe Medical Center, Israel; 5 Internal Medicine C, Hillel Yaffe Medical Center, Israel; 6 Pharmacy Department, Hillel Yaffe Medical Center, Israel; 7 Faculty of Health Sciences, Ben-Gurion University of the Negev, Be’er Sheva, Israel

## Abstract

**Background:**

Carbapenem-resistant *Acinetobacter baumannii* (CRAB) is a major challenge, yet the role of hospital-wide active surveillance cultures (ASC) outside outbreak settings remains debated. We assessed the impact of targeted ASC and cohorting integrated with an established infection prevention and control (IPC) program in an endemic setting on CRAB epidemiology.

**Methods:**

A retrospective study was conducted at a 515-bed university hospital in Israel (January 2016–August 2025; 2020–2021 excluded). Screening of high-risk patients commenced in January 2023 utilizing four-limb skin sponges, with comprehensive implementation and patient cohorting initiated in July 2023. Outcomes were CRAB present on admission (POA) and hospital-acquired CRAB (overall and clinical subsets). We fit quasi-Poisson ITS models (January 2022–August 2025) with log link and exposure offsets (admissions for POA; hospital days for acquired); total counts were modeled with negative binomial regression. Cases averted were estimated with rate-based and ITS counterfactuals.

**Results:**

POA detection increased markedly after screening implementation (median 0.72 to 2.98 per 1,000 admissions), revealing a large reservoir of admission carriage. Overall hospital-acquired CRAB rates showed no sustained decline. In contrast, hospital-acquired clinical-CRAB fell substantially after July 2023 (mean 3.93 to 0.97 per 10,000 hospital-days), with a marked post-intervention level reduction in the ITS model (Incidence-rate ratio (IRR) 0.224, 95% CI 0.071–0.072, *P* = .0103). Counterfactual analyses estimated approximately 60–70 hospital-acquired clinical CRAB cases averted during follow-up.

**Conclusions:**

In an endemic setting, hospital-wide ASC targeting high-risk patients unmasked extensive admission carriage and, combined with cohorting and other IPC measures, was associated with a substantial reduction in acquired clinical-CRAB cases.

## Introduction

Carbapenem-resistant *Acinetobacter baumannii* (CRAB) is one of the most challenging multidrug-resistant organisms (MDROs) encountered in hospital settings, characterized by extreme antimicrobial resistance, environmental persistence, and association with high morbidity and mortality, particularly in intensive care units (ICUs) and high-dependency rooms.^1^

The role of active surveillance cultures (ASC) among infection prevention and control (IPC) efforts remains uncertain.^2^ While ASC is often accepted for carbapenemase producing Enterobacterales (CPE), the scientific basis for its use is limited. A systematic review of ASC interventions for carbapenem-resistant Gram-negative bacteria found that most studies were conducted in outbreak settings, and although many reported reductions in colonization or infection rates, there was no effect on mortality or length of hospital stay, and the overall level of evidence was low.^3^ Only a small subset of studies in that review focused specifically on CRAB, and they did not involve hospital-wide screening or include effect of ASC on colonization rates.^4–6^ Concordantly, current guidelines like those issued by the World Health Organization^2^ and the ESCMID^7^—do not recommend routine CRAB-ASC outside of endemic or high-risk settings.

Further complicating interpretation of ASC effect is its frequent bundling with other IPC measures, making it difficult to isolate ASC-attributed impact. To date, only few studies have evaluated the implementation of CRAB-ASC as part of hospital-wide interventions.^8,9^ In those studies, screening methods were of relatively low sensitivity, and in at least one case, follow-up periods after ASC introduction on a wide scale were relatively short, and effect on colonization was not evaluated.^9^ This evidence gap is concerning given the rising burden of CRAB in acute-care hospitals.

The current study aims to address this gap by evaluating the effect of introduction of CRAB-ASC targeting high-risk patients, coupled with the cohorting of identified carriers. Outcomes assessed included both clinical infections and colonization dynamics over a multi-year period.

## Methods

### Settings

A retrospective cohort study was conducted at a 515-bed, university-affiliated hospital in Hadera, Israel. The hospital includes six high-dependency, open-space rooms across four internal medicine wards and two surgical wards; each room may house up to 4 ventilated patients. Regular rooms in these departments typically house 4 patients. Two internal medicine wards had 2 single-bed rooms, and the other two had 6.

### Data collection and definitions

All CRAB-positive screening and clinical culture results reported between January 2016 and August 2025 were retrieved from the microbiology laboratory’s computerized systems. The years 2020 and 2021 were excluded because of the COVID-19 pandemic and a cyberattack that occurred in late 2021 that resulted in loss of data. Each case was determined as being present on admission (POA) when CRAB was identified during the first 72 hours from admission (in a non-known carrier); otherwise, the case was determined as acquisition. Cases were also divided into clinical (identified by any non-screening culture) and screening cases. Patients who had both positive screening and clinical samples were classified as clinical cases. Positive cultures from known carriers were excluded.

### IPC settings and intervention

The infection control (IC) unit included one physician, 3 full-time nurses, and IC trustees across the hospital wards (dedicated mostly to hand hygiene (HH) and isolation monitoring). An existing IPC program included monitoring and reporting of the compliance of HH and of MDROs (CRAB, MRSA, VRE, CDI, *C. auris,* and CPE) isolation and personal protective equipment (PPE) usage, as well as manually flagging of MDROs-carriers in the computerized systems. An antimicrobial stewardship for restriction of wide-range antimicrobials was established across the hospital. Since June 2022, several new interventions have taken place. These included enhanced HH monitoring (increasing the number of interventional monitoring); introduction of enhanced routine cleaning protocols using 2000 ppm of bleach for MDROs-rooms; introduction of instantaneous automatic SMS messages reporting a laboratory identification of an MDRO and of new hospitalization of a MDRO-carrier, sent to the cellular phones of both the IC team and the corresponding head nurse of the recipient ward; introduction of daily, weekly, and monthly reports regarding the prevalence and new acquisition of MDROs; daily chlorhexidine-gluconate baths for patients located in the high-dependency rooms and in ICU wards, as well as for patients carrying central venous catheters; and the introduction of terminal cleaning protocol after patients’ discharge (established only in the 4 internal medicine wards).

Since January 2023, skin screening for CRAB was introduced for high-risk patients upon admission that included patients: (a) arriving from long-term care facilities (LTCFs)/other hospitals, (b) admitted to ICU/high-dependency rooms, (c) mechanically ventilated, or (d) transferred within the hospital. Weekly re-screening was conducted for hospitalized patients meeting these criteria. Screening used skin premoistened sponges on four limbs. Screening was established and was considered fully implemented since July 2023. Concurrently, from July 2023, a section of one internal medicine ward (4 rooms, up to 10 patients) was dedicated to CRAB cohort care, although dedicated staff was not consistently available throughout the years. Table 1 and Supplementary Figure 3B depict a timeline of interventions.

**Table 1.** Timeline of IPC program evolution^a^ Table 1 long description.

		2016	2017	2018	2019	2022	1–6/2023	7–12/2023	2024	1–8/2025
Hand hygiene	Number of audits	ND	ND	3,075	2,537	3,563	3,847^b^	6,189^b^	11,700	4,112^c^
	% compliance	75	75	82	89	79	82	80	89	84
Cleaning audits	Number of audits	ND	ND	98	814	135	378^b^	295^b^	349	305^c^
	% clean	ND	ND	ND	39	54	79	91	89	90
PPE & isolation of patients with MDROs	Number of audits	ND	198	43	392	50	42	53	57	34^c^
	% compliance	ND	88	97	98	97	93	98	98	97
Terminal cleaning (internal medicine wards only)	Number of audits	NR	NR	NR	NR	NR	NR	NR	71^d^	78^c^
	% clean	NR	NR	NR	NR	NR	NR	NR	94^d^	99^c^
Monthly reports of MDRO acquisitions		No	No	No	No	Yes^e^	Yes	Yes	Yes	Yes
Antibiotic stewardship		Yes	Yes	Yes	Yes	Yes	Yes	Yes	Yes	Yes
Flagging of MDROs in computerized systems		Manually	Manually	Manually	Manually	Automatic^e^	Automatic	Automatic	Automatic	Automatic
Using 2000 ppm bleach for MDROs routine cleaning		No	No	No	No	No^f^	Yes	Yes	Yes	Yes
SMS on identification/hospitalization of MDROs to wards		No	No	No	No	No	Yes^g^	Yes	Yes	Yes
CHG baths for high-risk patients		No	No	No	No	No	No	No	Yes^h^	Yes
CRAB screening		No	No	No	No	No	Initiated	Yes^i^	Yes	Yes
CRAB cohorting		No	No	No	No	No	No	Yes	Yes	Yes

ND, no data; NR, not relevant; CHG, chlorhexidine gluconate; MDRO, multidrug-resistant organism; PPE, personal protective equipment; CRAB, carbapenem resistant *A. baumannii.*

a Data related only to CRAB relevant wards, ^b^for only 6 months, ^c^for 8 only months, ^d^started on April 2024, ^e^started on June 2022, ^f^started only in November 2022, ^g^started on February 2023, ^h^started on October 2024, ^i^started on January 2023 but fully implemented in July 2023.

### Microbiological methods

Specimens were enriched overnight in BHI broth and cultured on modified CHROMagar MDR-Acinetobacter plates (Hylabs, Rehovot, Israel). Clinical isolates were identified by MALDI-TOF MS (Bruker Biotyper, MA, USA) and antimicrobial resistance determined by meropenem and imipenem disk diffusion susceptibility testing per CLSI guidelines.

The study was approved by the institutional review board (HYMC0167-24).

### Statistical analysis

Data from January 2016 through August 2025 were analyzed, excluding calendar years 2020–2021. Incidence was summarized as monthly counts and normalized to the relevant denominators, expressed as rates per 1,000 admissions for POA events and per 10,000 hospital-days for hospital-acquired events. Hospital-days comprised patient-days from four internal-medicine wards, two surgical wards, two orthopedic wards, neurology, urology, and two intensive-care units.

For descriptive purposes, mean and median monthly rates and overall incidence were calculated by study phase. Temporal associations were evaluated using interrupted time-series (ITS) regression with quasi-Poisson models (log link) and an exposure offset (log[admissions] for POA series; log[hospital-days] for acquired outcomes). Overdispersion was accommodated by the quasi-Poisson variance function.

Segmented ITS models were prespecified to assess changes across key implementation milestones, focusing on the recent period of January 2022–August 2025. For intervention-targeted outcomes (Figures 2A and B), we used a two-breakpoint segmented regression reflecting enhanced screening implementation (January 2023) and full implementation including cohorting (July 2023). To allow for an abrupt but potentially transient perturbation at the time of screening introduction, a January 2023 pulse (temporary-level change) indicator was included. The model was specified as:

$$\begin{array}{rl} Log\;E(Yt)= & \beta_{0}+\beta_{1}t+\beta_{2}I(t=\tau_{1})+\beta_{3}(t-\tau_{1}+1)\\ & +\beta_{4}I(t\geq \tau_{2})+\beta_{5}(t-\tau_{2}+1)+log(exposuret) \end{array}$$

where *Yt* is the monthly count, *t* is the month index, τ_1_ and τ_2_ denote January 2023 and July 2023, *I*() is an indicator function, (x)_+_ = max(x,0), and exposure_t_ is the monthly denominator.

In contrast, for the POA-clinical CRAB series (Figure 3A), which was not affected by the intervention, we prespecified a trend-only model (time and exposure offset, without breakpoint terms).

For acquired clinical CRAB (Figure 3B), we specified a single-break segmented ITS model in July 2023, as screening introduction could not plausibly affect hospital-acquired clinical cases, whereas the cohorting-based intervention could.

Incidence-rate ratios (IRRs) with 95% confidence intervals (CIs) were reported for baseline level and for level and slope changes at each intervention phase; segment-specific monthly slopes were derived from linear combinations of the relevant coefficients. Model-based fitted values were used to generate the plotted lines, and shaded ribbons represent pointwise 95% CIs.

Heteroskedasticity- and autocorrelation-consistent (HAC) covariance estimators (sandwich *vcovHAC*) provided robust standard errors and 95% CIs. As a sensitivity analysis, Newey–West standard errors (lag = 6) were also computed and yielded similar inference, supporting model stability. Residual autocorrelation was assessed using Durbin–Watson and Ljung–Box tests on Pearson residuals; HAC-based inference was retained throughout for robustness and consistency across models.

For acquired-clinical CRAB, we estimated cases averted using two complementary counterfactual approaches. First, we compared two epidemiologically stable periods—January–December 2022 (baseline) and July 2023–August 2025 (steady-state postimplementation), excluding the January–June 2023 transition—and calculated the expected number of postcohorting cases by applying the baseline rate to postcohorting hospital days. Second, using the single-break ITS model, we generated a model-based counterfactual in which the July-2023 level and slope were set to zero; predicted postintervention counts under this “no-bundle” scenario were summed and compared with the observed counts.

All analyses were conducted in R 4.4.0 (R Foundation, Vienna) using packages *MASS, lmtest, sandwich, dplyr, lubridate, zoo,* and *ggplot2*. ChatGPT (OpenAI, San Francisco, CA) was used as an interactive programming assistant to refine R code syntax and documentation; all analyses and model outputs were independently verified by the authors.

## Results

### CRAB burden

**Figure 1.**
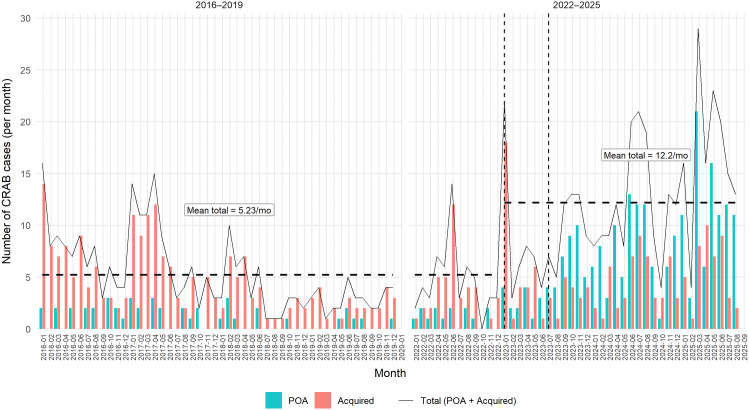
Figure 1 long description. Total CRAB burden (POA and acquired). Bars show observed monthly counts of CRAB cases classified as present on admission (POA) and hospital-acquired. The solid black line indicates the total monthly burden (POA + hospital-acquired). Vertical dashed lines mark January 2023 (screening introduction) and July 2023 (cohorting and full screening implementation). Dashed horizontal segments indicate the mean total monthly burden for January 2016–December 2022 and for January 2023–August 2025. Calendar years 2020–2021 were excluded. Mo, month; POA, present on admission.

During the preintervention period (January 2016 through December 2022), the mean total CRAB burden was 5.23 new cases per month (95% CI 4.31–6.16; median 4), of which 0.98 were POA and 4.25 were hospital-acquired. In the period after introduction of admission screening (January 2023–August 2025), the mean total CRAB burden increased to 12.2 cases per month (95% CI 9.91–14.4; median 12), driven almost entirely by a marked increase in POA detections (mean 7.41 per month), while hospital-acquired cases remained largely unchanged (mean 4.75 per month) (Figure 1).

### CRAB-POA corrected rates

**Figure 2.**
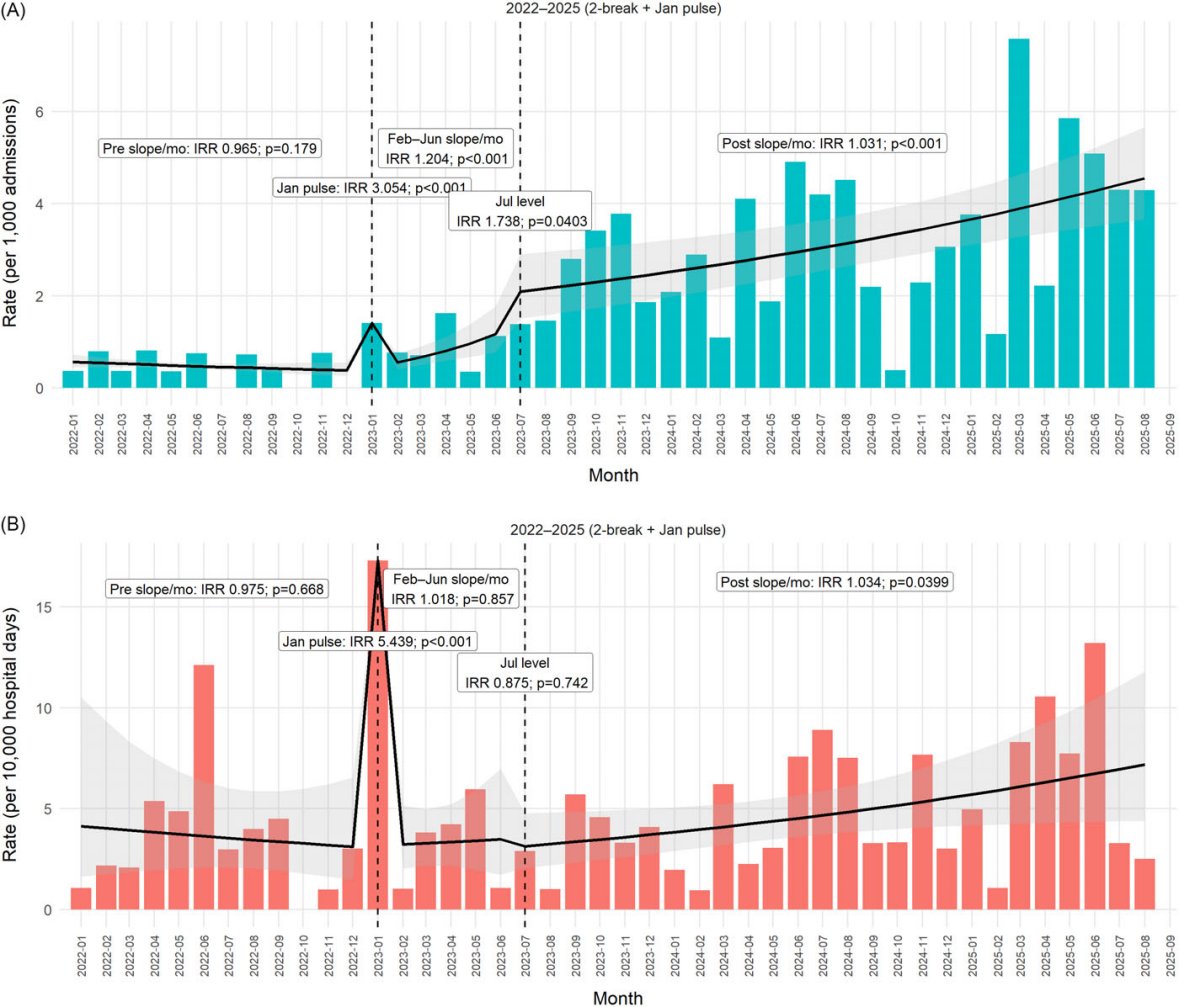
Figure 2 long description. CRAB POA vs acquisition. Monthly bars represent observed CRAB incidence rates per 1,000 admissions for CRAB present on admission (Figure 2A) and per 10,000 hospital-days for hospital-acquired CRAB (Figure 2B). The solid black line depicts the fitted trend over time, with the shaded area indicating the corresponding confidence interval. Vertical dashed lines mark January 2023 (screening introduction) and July 2023 (cohorting and full screening implementation). Annotated labels indicate estimated rate ratios for changes in slope or level across time segments, including a transient January 2023 pulse. Mo, month; Jan, January; Feb, February; Jun, June; Jul, July; IRR, incidence-rate ratio; POA, present on admission.

Preintervention, POA-corrected rates showed no significant temporal trend (preintervention slope IRR/month 0.965, 95% CI 0.916–1.016; *P* = .179), Figure 2A. The initiation of enhanced screening in January 2023 was associated with a large transient increase in detected POA carriage (pulse IRR 3.05, 95% CI 2.20–4.23; *P* < .001). During the subsequent February–June 2023 transient period, POA rates continued to rise (slope IRR/month 1.20, 95% CI 1.10–1.32; *P* < .001). Following full implementation of the intervention bundle in July 2023, there was an additional level increase in POA detection (IRR 1.74, 95% CI 1.03–2.95; *P* = .04), together with a sustained positive postintervention slope (IRR/month 1.031, 95% CI 1.014–1.049; *P* < .001). Consistent with these model-based estimates, the observed POA rate increased from a median of 0.72 per 1,000 admissions (mean 0.63) before July 2023 to 2.98 per 1,000 admissions (mean 3.18) thereafter.

### Acquired-CRAB-corrected rates

Before the introduction of active screening, acquired CRAB rates showed no underlying secular trend (preJanuary 2023 slope IRR per month 0.97, 95% CI 0.87–1.10; *P* = .67), Figure 2B. In January 2023, coincident with the initiation of systematic screening and isolation, a pronounced one-month detection spike was observed (pulse IRR 5.44, 95% CI 3.02–9.78; *P* < .0001), consistent with abrupt uncovering of previously undetected colonization rather than a true transmission surge. During the subsequent partial-implementation phase (February–June 2023), no significant change in the underlying acquisition trend was detected (slope IRR per month 1.02, 95% CI 0.84–1.24; *P* = .86). The transition to the full infection-control bundle in July 2023 was not associated with an immediate level change (IRR 0.88, 95% CI 0.40–1.94; *P* = .74). However, a small but statistically significant upward slope was observed thereafter (post July 2023 slope IRR per month 1.034, 95% CI 1.002–1.067; *P* = .040). Observed acquired rates increased modestly from a median of 2.59 per 10,000 hospital-days (mean 3.84) before July 2023 to 3.71 per 10,000 hospital-days (mean 4.96) thereafter.

### POA-clinical CRAB corrected rates

**Figure 3.**
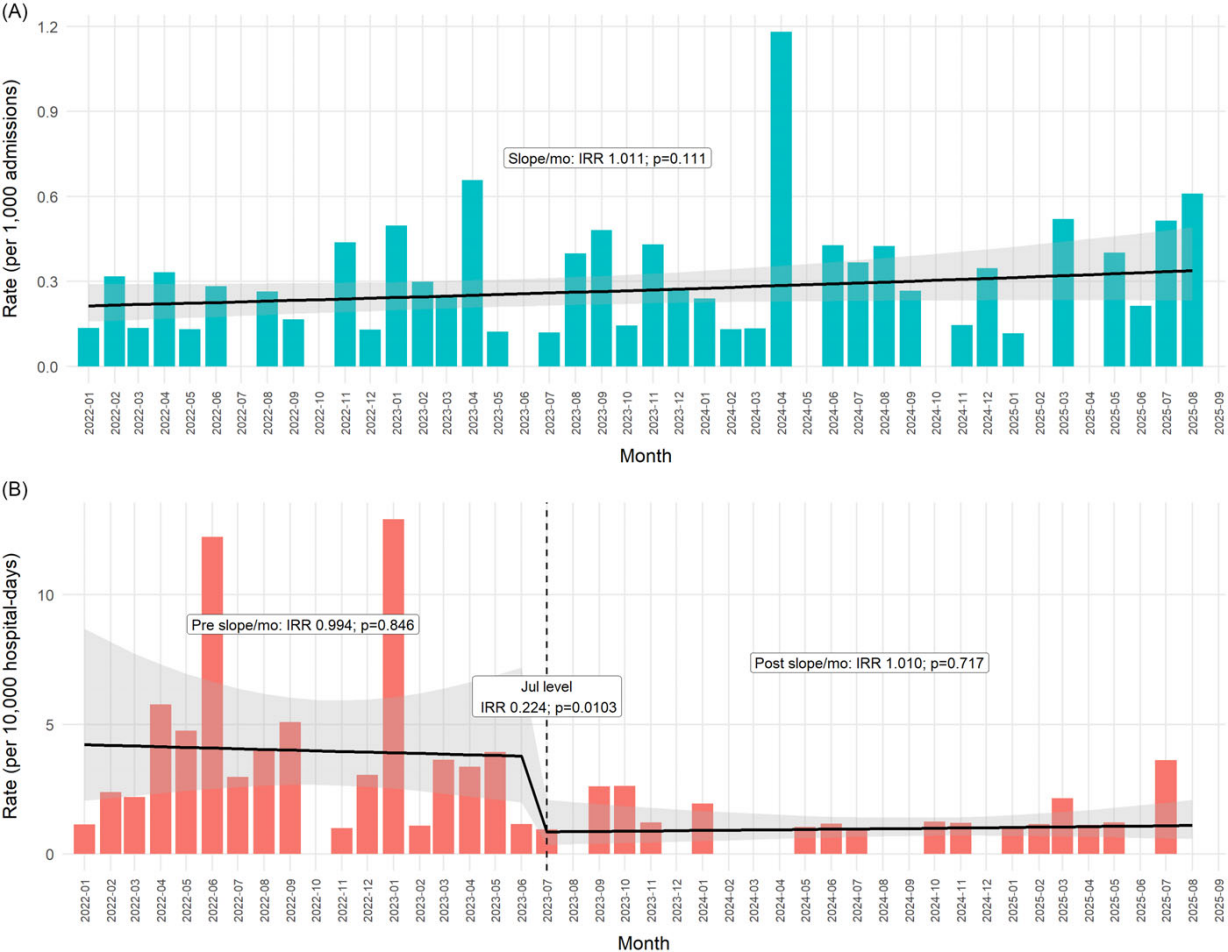
Figure 3 long description. CRAB clinical rates—POA vs acquisition. Monthly bars represent observed incidence rates of clinical CRAB per 1,000 admissions for cases present on admission (Figure 3A) and per 10,000 hospital-days for hospital-acquired cases (Figure 3B). The solid black line depicts the fitted trend over time, with the shaded area indicating the corresponding confidence interval. In Figure 3B, the vertical dashed line marks July 2023 (cohorting and full screening implementation). Annotated labels indicate estimated incidence-rate ratios for changes in slope or level across time segments. Mo, month; Jul, July; IRR, incidence-rate ratio; POA, present on admission.

POA-clinical CRAB rates exhibited a modest upward trend over time (IRR per month 1.011, 95% CI 0.998–1.024; *P* = .11), which did not reach statistical significance, Figure 3A. Median monthly POA-clinical rates were similar before and after July 2023 (0.151 vs 0.272 per 1,000 admissions), and no abrupt level change was observed. Because admission screening and in-hospital cohorting cannot influence the occurrence of true imported clinical infections, this series was modeled using a trend-only ITS framework without intervention breakpoints.

### Acquired-clinical CRAB-corrected rates

Prior to July 2023, acquired-clinical CRAB rates showed no underlying time trend (preintervention slope IRR per month 0.994, 95% CI 0.93–1.06; *P* = .846), Figure 3B. At the time of full bundle implementation in July 2023, a large and statistically significant immediate reduction was observed (level IRR 0.224, 95% CI 0.071–0.702; *P* = .010). Following this intervention, the monthly trend remained flat (post July slope IRR per month 1.010, 95% CI 0.96–1.07; *P* = .717). The median monthly acquired-clinical rate declined from 3.21 per 10,000 hospital-days before July 2023 to 1.02 thereafter, with corresponding means of 3.93 and 0.97, respectively.

### Acquired-clinical CRAB cases averted

When comparing the stable preintervention period (January 2022–December 2022) with the steady-state postimplementation period (July 2023–August 2025), hospital-acquired clinical CRAB rates declined markedly. A Poisson regression with hospital days as an offset and HAC standard errors showed a 74% relative reduction in incidence after full implementation (IRR 0.26, 95% CI 0.14–0.48, *P* < .0001). Results were nearly identical using a negative binomial model (IRR 0.26, 95% CI 0.14–0.48, *P* < .0001). Applying the preintervention acquired-clinical CRAB rate to the postintervention exposure yielded ∼ 91 expected cases, compared with 21 observed, corresponding to ∼ 70 hospital-acquired clinical CRAB infections averted (95% CI 47.2–78.4). Consistent with this estimate, a July-2023 ITS counterfactual model predicted 84.3 postintervention cases, corresponding to ∼ 61 acquired-clinical CRAB infections averted.

## Discussion

In this single-center ITS analysis, the stepwise implementation of screening and cohorting within an established infection-prevention framework was associated with two distinct and complementary signals. Introduction of a sensitive four-limb screening method in January 2023 was followed by a marked increase in detection of CRAB on admission, consistent with improved ascertainment of a large CRAB reservoir, illustrating the tip-of-the-iceberg principle. Second, full implementation of the intervention bundle in July 2023 was associated with a sustained and statistically significant decline in hospital-acquired clinical CRAB, with a level reduction of approximately 78% that persisted across the 26-month period. Counterfactual analyses indicate that approximately 60–70 acquired-clinical CRAB cases were averted during follow-up.

These results align with prior Israeli experience demonstrating that only large-scale screening with cohorting can reduce hospital-acquired CRAB in endemic settings.^8^ Our contribution extends this work by explicitly disentangling POA from acquired events, distinguishing clinical from screen-detected outcomes, and applying formal segmented regression to separate the effects of partial screening and full screening and cohorting added on an established IPC program. Importantly, we employed a four-limb skin swab method,^10,11^ which is more sensitive than single-site or rectal sampling and likely enhanced ascertainment of carriage at admission. Unlike other studies that also used skin or peri-rectal screening to mitigate ICU outbreaks,^12–15^ we implemented the screening broadly across all high-risk wards. Given the widespread endemicity of CRAB outside Israel,^16^ integrating ASC and cohorting into IPC programs should be more widely considered.

The most significant effect of the intervention was the marked reduction of hospital-acquired clinical CRAB cases. The recurring outbreak pattern of acquired CRAB cases observed before the intervention, predominantly driven by clinical cases—collapsed after July 2023 (Figures 1, 3B). During the 26-month postcohorting period, only 21 acquired clinical cases were observed (13 in internal medicine wards, 3 in the ICU, and 5 in surgical wards). Of these, 7 were bloodstream infections, while the remainder were clinical isolates from respiratory, urine, or wound cultures that may not all have represented invasive disease. Based on the 90-day mortality rates among patients with hospital-acquired clinical CRAB in this cohort,^17^ averting 60–70 clinical CRAB cases would be expected to translate into roughly ∼ 40 fewer deaths, in addition to substantial reductions in morbidity and healthcare burden.

The intriguing selective reduction in the acquisition of clinical CRAB cases is likely to be explained by two complementary mechanisms: (i) early identification of POA carriers along with cohorting and other IPC measures enabled isolation that substantially reduced a massive, previously undetected, cross-transmission within the hospital, especially in high-dependency rooms where fragile patients are prone to develop clinically overt and detrimental infection; and (ii) prevention of progression to clinical disease among residual acquisitions detected presymptomatically by surveillance.

The implementation of ASC uncovered a large reservoir of CRAB carriage which more than doubled the monthly number of identified carriers and frequently exceeded the capacity of the dedicated 10-bed CRAB cohort. As a result, carriers were often placed in single rooms across multiple wards and transferred to the cohort when feasible. These challenges were compounded when patients carried additional MDROs requiring different isolations (e.g., CPE), further increasing bed-management complexity. New detections during hospitalization required relocation between wards, increasing workload and logistical strain. In addition, a dedicated cohorting team could not be maintained consistently, and the physical location of the cohort—at the periphery of ward—limited access to specialized care for these complex or ventilated patients (demographic data on these patients were published in^17^). Together, these real-world constraints likely sustained a background of low-level acquisition even as outbreak surges of clinical CRAB disappeared. Because CRAB carriage can progress to clinical infection^18^ and hospital-acquired clinical CRAB is associated with high mortality in this cohort,^17^ further reducing residual screen-detected acquisition remains an important target for ongoing program optimization. Finally, the program incurred substantial ongoing financial costs related to laboratory testing (∼ 25,640€/year), isolation resources, staffing, and infrastructure.

### Limitations

This single-center, quasi-experimental study is vulnerable to secular trends and co-interventions that ITS cannot fully eliminate. The effect of ASC and cohorting observed in this study may not be reproduced in facilities with different ward designs, e.g., facilities without four-bed rooms or high-dependency rooms for ventilated patients outside of the ICU. While we used quasi-Poisson with HAC for rate outcomes and negative binomial for counts to address overdispersion and serial correlation, residual autocorrelation, seasonality, or nonlinearity may remain. Multiple IPC measures were taken during the study period, and although ASC and cohorting were chosen as breakpoints based on temporal fluctuation in CRAB epidemiology, synergistic contributions are highly plausible. Molecular typing was not performed and would strengthen inference about transmission chains. Finally, generalizability depends on local epidemiology including CRAB prevalence, LTCF inflow, strain landscape, and IPC resources.

## Conclusions

In an endemic, high-burden setting, admission surveillance revealed a large reservoir of CRAB on admission and, when combined with cohorting and enhanced IPC measures, was associated with an approximately 78% reduction in hospital-acquired clinical CRAB. Hospitals with similar epidemiology should consider targeted admission screening of high-risk patients (notably LTCF transfers and patients requiring mechanical ventilation), paired with cohorting—ideally with dedicated staff—and continued investment in rapid diagnostics and isolation capacity. Even under real-world constraints, integrating this intervention within a bundled IPC program can substantially reduce CRAB transmission and its clinical burden.

## Supporting information

Cohen et al. supplementary material Cohen et al. supplementary material

## Supplementary material

The supplementary material for this article can be found at https://doi.org/10.1017/ice.2026.10427.

## Figure 1 Long description

The line graph illustrates the monthly counts of CRAB cases from January 2016 to August 2025. The x-axis represents the months, while the y-axis shows the number of CRAB cases per month. The graph includes two sets of bars: teal bars for POA cases and red bars for acquired cases. A solid black line represents the total monthly burden of CRAB cases, which is the sum of POA and acquired cases. Vertical dashed lines mark significant dates: January 2023 for the introduction of screening and July 2023 for the implementation of cohorting and full screening. Dashed horizontal lines indicate the mean total monthly burden for two periods: January 2016 to December 2022 and January 2023 to August 2025. The mean total monthly burden for the first period is approximately 5.23 cases, while for the second period, it is approximately 12.2 cases. The graph shows fluctuations in the number of cases over time, with notable increases after the implementation of screening and cohorting. All values are approximated.

Navigate back to Figure 1.

## Figure 2 Long description

Two bar graphs with overlaid line graphs show CRAB incidence rates from January 2022 to September 2025. The first graph (Figure 2A) displays monthly bars representing observed CRAB incidence rates per 1,000 admissions for CRAB present on admission. The second graph (Figure 2B) shows monthly bars representing observed CRAB incidence rates per 10,000 hospital-days for hospital-acquired CRAB. Both graphs feature a solid black line depicting the fitted trend over time, with a shaded area indicating the corresponding confidence interval. Vertical dashed lines mark January 2023 and July 2023, indicating the introduction of screening and the implementation of cohorting and full screening, respectively. Annotated labels indicate estimated rate ratios for changes in slope or level across time segments, including a transient January 2023 pulse. The first graph shows a pre-slope of 0.965, a February to June slope of 1.204, and a post-slope of 1.031. The second graph shows a pre-slope of 0.975, a February to June slope of 1.018, and a post-slope of 1.034. All values are approximated.

Navigate back to Figure 2.

## Figure 3 Long description

The image contains two line graphs. The first graph (Figure 3A) shows the monthly incidence rates of clinical CRAB per 1,000 admissions for cases present on admission. The second graph (Figure 3B) shows the monthly incidence rates of clinical CRAB per 10,000 hospital-days for hospital-acquired cases. The solid black line in both graphs depicts the fitted trend over time, with the shaded area indicating the corresponding confidence interval. In Figure 3B, the vertical dashed line marks July 2023, indicating the implementation of cohorting and full screening. Annotated labels indicate estimated incidence-rate ratios for changes in slope or level across time segments. The x-axis represents the months from January 2022 to September 2025, and the y-axis represents the rate per 1,000 admissions in Figure 3A and the rate per 10,000 hospital-days in Figure 3B. All values are approximated.

Navigate back to Figure 3.

## Table 1 Long description

The table presents the evolution of infection prevention and control program interventions from 2016 to 2025. It includes data on hand hygiene, cleaning audits, personal protective equipment and isolation of patients with multidrug-resistant organisms, terminal cleaning of internal medicine wards, monthly reports of multidrug-resistant organism acquisitions, antibiotic stewardship, flagging of multidrug-resistant organisms in computerized systems, use of bleach for routine cleaning, short message service on identification and hospitalization of multidrug-resistant organisms to wards, chlorhexidine gluconate baths for high-risk patients, carbapenem-resistant Acinetobacter baumannii screening, and carbapenem-resistant Acinetobacter baumannii cohorting. The table has eight rows and nine columns, with column headers for each year from 2016 to 2025 and row labels for each intervention category. Notable trends include the introduction of skin screening for carbapenem-resistant Acinetobacter baumannii for high-risk patients upon admission since January 2023 and the full implementation of this screening since July 2023. Weekly re-screening was conducted for hospitalized patients meeting specific criteria. The table also shows the dedication of a section of one internal medicine ward to carbapenem-resistant Acinetobacter baumannii cohort care since July 2023, although dedicated staff was not consistently available throughout the years.

Navigate back to Table 1.
