# Supplementary figures and images for: Active surveillance cultures and cohorting for carbapenem-resistant Acinetobacter baumannii in an endemic setting: an interrupted time-series analysis
Source: Infect Control Hosp Epidemiol. 2026 Apr 1;47(6):586–92. doi: 10.1017/ice.2026.10427 (PMC13216810; doi:10.1017/ice.2026.10427)

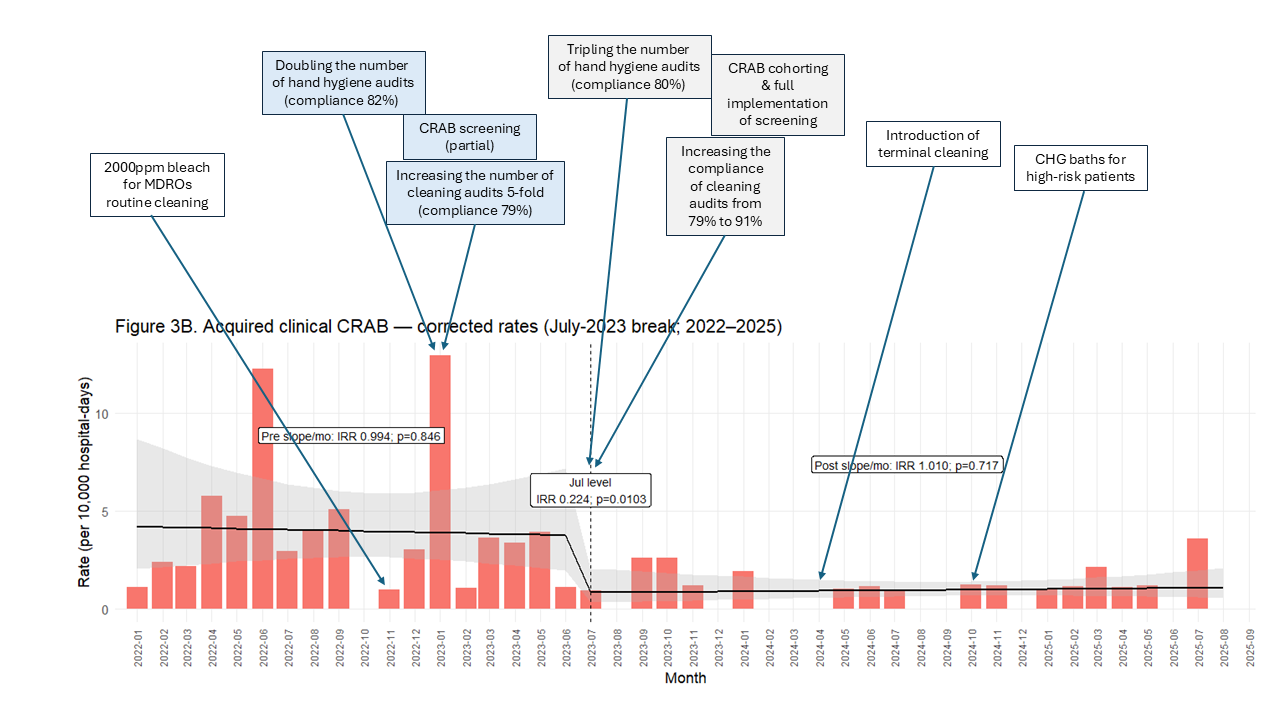

Supplement: Cohen et al. supplementary material [file S0899823X26104279sup001.tiff]
